# Navigating the Complexities of Microneurosurgery Practice in Iraq

**DOI:** 10.7759/cureus.71972

**Published:** 2024-10-20

**Authors:** Ali Alshalchy, Sajjad G Al-Badri, Rania H Al-Taie, Mustafa Ismail

**Affiliations:** 1 Department of Neurosurgery, University of Baghdad, College of Medicine, Baghdad, IRQ; 2 Department of Surgery, University of Baghdad, College of Medicine, Baghdad, IRQ; 3 Department of Surgery, University of Mustansiriyah, College of Medicine, Baghdad, IRQ; 4 Department of Surgery, Baghdad Teaching Hospital, Medical City Complex, Baghdad, IRQ

**Keywords:** educational barriers, healthcare infrastructure, international collaboration, microneurosurgery, political instability

## Abstract

Microneurosurgery in Iraq confronts numerous obstacles stemming from educational limitations, technological deficits, political instability, financial constraints, cultural challenges, and regulatory hurdles. The scarcity of specialized training programs and outdated educational opportunities impede the cultivation of neurosurgical expertise, often compelling aspiring surgeons to seek inaccessible training abroad due to financial and geopolitical barriers. A lack of advanced medical technology and equipment, worsened by funding shortages and import restrictions, hampers surgeons' ability to perform complex procedures safely and effectively. Prolonged political instability has devastated healthcare infrastructure and spurred a brain drain, further diminishing the nation's neurosurgical capabilities. Cultural and societal factors, such as skepticism toward advanced surgical interventions and tribal decision-making processes, lead to patient reluctance and additional pressures on medical professionals. Regulatory and administrative challenges complicate the importation of essential equipment and the recognition of international qualifications. Addressing these multifaceted issues necessitates coordinated efforts from the Iraqi government, medical institutions, and international partners to invest in education, modernize healthcare infrastructure, enact policy reforms, and foster global collaboration, thereby enhancing the practice of microneurosurgery in Iraq.

## Editorial

Background

Microneurosurgery is one of the most advanced and intricate fields within modern medicine, demanding highly specialized skills and access to cutting-edge technology. The practice of microneurosurgery, however, presents significant challenges in developing countries like Iraq, where political instability, limited financial resources, and inadequate healthcare infrastructure persist. The pathway to becoming a neurosurgeon is arduous, and for those practicing in Iraq, the lack of resources and educational support makes it even more daunting. In this editorial, we will explore the multifaceted challenges facing microneurosurgery in Iraq, including educational barriers, technological deficits, and societal issues, while emphasizing the need for systemic reforms to improve neurosurgical care in the region [1].

Overcoming these challenges requires concerted efforts at both national and international levels. Investment in education, modernization of medical infrastructure, and collaboration with global medical communities are essential to improving neurosurgical practices in Iraq. The importance of this issue cannot be overstated, as access to advanced neurological care is critical for treating life-threatening conditions such as brain tumors, aneurysms, and traumatic brain injuries. Without proper interventions, many patients face dire outcomes. The time is ripe for a renewed focus on building the capacity for microneurosurgery in Iraq, a vital component of healthcare that has been long overlooked [2].

The foundation of modern neurosurgery in Iraq dates to the 1950s, when Dr. Najeeb Al-Yaaqubi, a general surgeon, performed the nation's first elective neurosurgical operation. However, a formal neurosurgical practice wasn't established until 1966, driven by pioneers like Dr. Saad Al-Witry, who was celebrated as the founder of neurosurgery in Iraq. The first neurosurgical training program began in 1972 with the opening of the Neurosurgery Teaching Hospital (NTH) in Baghdad, a tertiary center featuring 150 beds and seven operating rooms. Today, NTH is staffed by 16 neurosurgeons, including four consultants. Importantly, modern microneurosurgery techniques were introduced only after 2003; before that, all surgeries were conducted without microscopic assistance [3].

Limited educational opportunities

One of the most significant obstacles to the development of neurosurgery in Iraq is the lack of specialized training programs. Aspiring neurosurgeons often have limited opportunities for advanced education and training within the country. Most available neurosurgery training programs are either too basic or outdated, lacking the depth required for advanced specializations like microneurosurgery. As a result, many neurosurgeons are forced to seek training abroad. However, studying in foreign countries is often out of reach due to financial constraints, geopolitical tensions, and visa issues. These barriers not only limit the number of fully trained neurosurgeons in the country but also prevent Iraq from building a robust neurosurgical community capable of advancing the field domestically [2].

Despite efforts to establish neurosurgical training facilities, such as the creation of Iraq’s first neurosurgery lab in Baghdad, these programs remain insufficient to meet the growing demand. Moreover, the lack of local fellowship programs and continuing professional development initiatives further stifles the growth of the field. Without opportunities for mentorship and hands-on learning in advanced neurosurgical techniques, Iraqi neurosurgeons struggle to remain up-to-date with the latest innovations. This gap in education has long-term implications for the quality of neurosurgical care available in the country, perpetuating a cycle of underdevelopment and professional stagnation [2].

Insufficient access to advanced technology

Advanced technology plays a crucial role in modern microneurosurgery, where precise, minimally invasive techniques are often required to perform delicate operations on the brain and nervous system. Unfortunately, many hospitals in Iraq lack access to the necessary tools, such as operating microscopes, neuronavigation systems, and high-resolution imaging devices. These tools are essential for surgeons to perform complex procedures with accuracy and safety. However, due to financial constraints and bureaucratic import restrictions, acquiring and maintaining this equipment remains a significant challenge. The absence of such technology not only limits the surgical capabilities of neurosurgeons but also places patients at risk for suboptimal outcomes [3].

Even when equipment is available, its maintenance and the consistent supply of necessary materials are often unreliable. Many hospitals face shortages of basic medical supplies, leaving healthcare professionals to make difficult decisions about resource allocation. This lack of advanced technology is further compounded by insufficient infrastructure, including outdated operating rooms and diagnostic facilities. In many cases, neurosurgeons are forced to work with inadequate tools in environments that are not conducive to modern surgical practices. This situation further discourages patients from seeking advanced neurosurgical care, eroding trust in the healthcare system and exacerbating the healthcare gap in Iraq [1].

Impact of political instability

The long history of political instability in Iraq has had a devastating impact on its healthcare system. Years of conflict, including wars, sanctions, and internal unrest, have left the country's medical infrastructure in a state of disrepair. Hospitals have been destroyed or rendered nonfunctional, and the healthcare workforce has been decimated by violence and mass emigration. In such an environment, it is difficult to attract the international investment and collaboration necessary to develop specialized medical fields like microneurosurgery. The instability also poses a direct threat to healthcare professionals, who face personal risks when working in conflict zones or politically volatile regions [4].

Political instability in Iraq has also exacerbated the influence of tribalism in medical decision-making, particularly in rural areas where traditional societal structures hold significant power. In many cases, patient care decisions are made not by the individuals themselves but by their extended families or tribes. This creates a unique set of challenges for neurosurgeons, who may face threats or demands for financial compensation if complications arise during or after surgery. The stress of navigating these social dynamics, combined with the pressures of working in an unstable political environment, drives many neurosurgeons to leave the country, further weakening Iraq’s healthcare system [2].

Brain drain phenomenon

One of the most significant consequences of Iraq's political and social instability is the exodus of skilled medical professionals, a phenomenon known as brain drain. Many of Iraq’s most talented neurosurgeons have left the country in search of safer, more stable working environments where they have access to the resources they need to provide high-quality care. This brain drain has severely depleted the ranks of experienced neurosurgeons in Iraq, leaving the country with a critical shortage of qualified professionals capable of performing complex neurological surgeries. This shortage has profound implications for patient care, as those requiring specialized interventions may have to wait long periods or travel abroad to receive treatment [5].

Brain drain also affects the development of future generations of neurosurgeons. With fewer experienced professionals remaining in Iraq, there are limited opportunities for mentorship and the transfer of knowledge to younger doctors. This perpetuates the cycle of underdevelopment in neurosurgical education and practice as the country struggles to train enough specialists to meet the needs of its population. Addressing the brain drain phenomenon requires not only improving working conditions and safety for healthcare professionals but also creating an environment that fosters professional growth and development within Iraq [1].

Financial constraints and limited funding

Financial constraints represent one of the most pressing challenges to the development of microneurosurgery in Iraq. Healthcare funding is severely limited, with government budgets often directed toward addressing more immediate public health concerns such as infectious diseases and trauma care. Specialized fields like neurosurgery receive a fraction of the funding needed to maintain high standards of care. As a result, hospitals are often unable to invest in the infrastructure, technology, and training programs necessary to support microneurosurgical practice. This lack of funding creates a vicious cycle where underinvestment in healthcare leads to poor outcomes, further diminishing public trust in the system and making it more difficult to secure future investments [4].

In addition to insufficient government funding, Iraq's healthcare sector faces challenges in securing international aid and investment. The country’s political instability and complex regulatory environment make it a risky proposition for international donors and investors. Without significant changes to the healthcare funding model, it will be difficult for Iraq to build the necessary infrastructure to support advanced medical fields like microneurosurgery. This situation underscores the importance of comprehensive policy reforms aimed at increasing financial support for specialized medical services, both from domestic and international sources [3].

Cultural and societal factors

Cultural and societal factors also play a significant role in the practice of microneurosurgery in Iraq. Many patients, particularly in rural areas, hold deep-seated skepticism toward advanced surgical procedures. This skepticism is often rooted in a lack of understanding about modern medical practices as well as traditional beliefs that prioritize family and tribal decision-making over individual autonomy. In many cases, patients may delay seeking treatment or refuse surgery altogether, which can lead to worsening outcomes and further strain on the healthcare system. Public education campaigns are needed to improve awareness of neurosurgical treatments and their potential benefits, helping to build trust in the healthcare system [1].

Moreover, neurosurgeons in Iraq face significant risks due to the cultural emphasis on tribalism. In cases where surgical complications arise, families may demand financial compensation or resort to violence against medical staff. These societal pressures make the already high-stakes field of neurosurgery even more challenging. Neurosurgeons must not only contend with the technical difficulties of their profession but also navigate a complex web of cultural expectations and interpersonal dynamics. This societal environment discourages many from pursuing careers in neurosurgery and contributes to the broader issue of brain drain within the medical community [1].

Lack of international collaboration

International collaboration is a critical component in advancing medical fields, particularly in developing countries where resources and expertise are limited. Unfortunately, Iraq's involvement in the global neurosurgical community has been minimal, largely due to political instability, financial constraints, and a lack of infrastructure to support international partnerships. This isolation prevents Iraqi neurosurgeons from accessing the latest innovations in surgical techniques, technology, and best practices, which are crucial for providing high-quality care to patients. Fostering international collaborations could help bridge these gaps and provide Iraqi neurosurgeons with the support they need to advance the field of microneurosurgery [5].

Encouraging partnerships with global medical institutions would offer several benefits, including access to cutting-edge technologies, opportunities for professional development, and the ability to participate in international research. Such partnerships would also facilitate the exchange of knowledge and skills, allowing Iraqi neurosurgeons to stay current with global best practices. By engaging in these collaborations, Iraq could enhance its healthcare system's ability to deliver specialized care. However, without the infrastructure to support such initiatives or the political will to prioritize healthcare reforms, the country risks falling further behind in the medical field, limiting the potential benefits of international cooperation [1].

Regulatory and administrative challenges

Bureaucratic hurdles and outdated regulatory frameworks are additional barriers to the growth of microneurosurgery in Iraq. Importing advanced medical equipment and technologies is a complex process fraught with delays due to inefficiencies in government systems. These delays make it difficult for hospitals to acquire and maintain the tools necessary for modern surgical procedures. Furthermore, the lack of streamlined processes for establishing new training programs or adopting innovative surgical techniques hampers the overall development of the medical field. Addressing these regulatory issues is crucial for creating a conducive environment for microneurosurgical advancement in Iraq [1].

Moreover, the administrative challenges extend to licensing and certification processes for neurosurgeons. Many Iraqi neurosurgeons who train abroad face difficulties in obtaining recognition for their qualifications when they return home. This limits their ability to practice at the highest levels and discourages others from seeking specialized training outside the country. Simplifying these processes and updating the regulatory framework to align with international standards would enable more Iraqi neurosurgeons to contribute meaningfully to the healthcare system. Additionally, reducing bureaucratic inefficiencies would encourage greater participation in international collaborations and increase the flow of medical innovations into Iraq [4].

Call to action

The neurosurgical care system in Iraq faces critical challenges that demand immediate attention. There is an urgent need for increased healthcare funding, particularly in microneurosurgery, and for policy reforms that enhance infrastructure and patient care. Establishing international partnerships with academic institutions can facilitate essential training and resource sharing. The future of neurosurgery in Iraq hinges on collaboration among the government, healthcare providers, and the global medical community, emphasizing investments in education, infrastructure, and international cooperation.

To address these challenges effectively, flexible regulations must be implemented to allow neurosurgery residents and surgeons to pursue fellowships abroad. International aid usually comes in two forms: providing training and mentorship to establish global connections and offering grant funding to initiate local practices or research initiatives. Simultaneously, both the governmental and private sectors within Iraq must streamline processes to create a more adaptable environment conducive to the seamless practice of microneurosurgery. By empowering institutions at the hospital level and expanding efforts to towns, cities, and eventually the entire country.

Conclusion

Microneurosurgery in Iraq faces significant challenges across educational, technological, political, and societal sectors. Overcoming these requires a coordinated effort involving the government, medical institutions, and the international community. Key actions include increased investment in education, healthcare infrastructure modernization, and global partnerships. While these challenges are not insurmountable, they demand urgent action to build capacity, improve medical training, and enhance access to technology. The international medical community should support these efforts through resource sharing and expertise. With the right interventions, Iraq can develop a sustainable healthcare system capable of delivering advanced neurosurgical care.
